# A systematic review and meta-analysis on impact of suboptimal use of antidepressants, bisphosphonates, and statins on healthcare resource utilisation and healthcare cost

**DOI:** 10.1371/journal.pone.0269836

**Published:** 2022-06-29

**Authors:** Kyu Hyung Park, Leonie Tickle, Henry Cutler

**Affiliations:** 1 Macquarie Business School, Macquarie University, North Ryde, New South Wales, Australia; 2 Macquarie University Centre for the Health Economy, North Ryde, Australia; Qatar University, QATAR

## Abstract

**Background:**

Depression, osteoporosis, and cardiovascular disease impose a heavy economic burden on society. Understanding economic impacts of suboptimal use of medication due to nonadherence and non-persistence (non-MAP) for these conditions is important for clinical practice and health policy-making.

**Objective:**

This systematic literature review aims to assess the impact of non-MAP to antidepressants, bisphosphonates and statins on healthcare resource utilisation and healthcare cost (HRUHC), and to assess how these impacts differ across medication classes.

**Methods:**

A systematic literature review and an aggregate meta-analysis were performed. Using the search protocol developed, PubMed, Cochrane Library, ClinicalTrials.gov, JSTOR and EconLit were searched for articles that explored the relationship between non-MAP and HRUHC (i.e., use of hospital, visit to healthcare service providers other than hospital, and healthcare cost components including medical cost and pharmacy cost) published from November 2004 to April 2021. Inverse-variance meta-analysis was used to assess the relationship between non-MAP and HRUHC when reported for at least two different populations.

**Results:**

Screening 1,123 articles left 10, seven and 13 articles on antidepressants, bisphosphonates, and statins, respectively. Of those, 27 were rated of good quality, three fair and none poor using the Quality Assessment Tool for Observational Cohort and Cross-Sectional Studies. In general, non-MAP was positively associated with HRUHC for all three medication classes and most prominently for bisphosphonates, although the relationships differed across HRUHC components and medication classes. The meta-analysis found that non-MAP was associated with increased hospital cost (26%, p = 0.02), outpatient cost (10%, p = 0.01), and total medical cost excluding pharmacy cost (12%, p<0.00001) for antidepressants, and increased total healthcare cost (3%, p = 0.07) for bisphosphonates.

**Conclusions:**

This systematic literature review is the first to compare the impact of non-MAP on HRUHC across medications for three prevalent conditions, depression, osteoporosis and cardiovascular disease. Positive relationships between non-MAP and HRUHC highlight inefficiencies within the healthcare system related to non-MAP, suggesting a need to reduce non-MAP in a cost-effective way.

## Introduction

Poor medication adherence or persistence (MAP) is related to increased morbidity and mortality [1–4] and greater healthcare resource utilisation and healthcare cost (HRUHC) [5–7]. Interventions to improve MAP are reported to give positive effects on morbidity, HRUHC and patient satisfaction [8, 9].

Suboptimal use of medication due to nonadherence and non-persistence (non-MAP) is prevalent in chronic conditions [10–12] because a long-term therapy is often interrupted by undesirable medication use, including erratic use, under-use and premature discontinuation of therapy. The prevalence of non-MAP across all chronic conditions has been estimated at approximately 50% by the World Health Organisation [10]. The annual cost of non-MAP to the US healthcare system has been estimated at between USD 100 billion and USD 289 billion [11, 13, 14].

Depression, osteoporosis, and cardiovascular disease (CVD) are among the most prevalent conditions in developed countries [15–17] and impose a large health and economic burden on society [18–21]. For example, in the US, the total annual economic burden of major depressive disorder was estimated at $210 billion [20]; total annual healthcare spending associated with osteoporosis fractures among Medicare beneficiaries at $57 billion [22]; and total annual healthcare system cost for heart disease or stroke at $214 billion [23]. In 2015 in Australia, the three disease groups, CVD, musculoskeletal conditions and mental and substance use disorders, accounted for around 39% of the total burden of disease as measured using disability-adjusted life year (DALY) [21]. Although MAP for these conditions is important in achieving clinical goals [16, 24, 25], reported MAP is relatively low [16, 26, 27].

Antidepressants, bisphosphonates, and statins are medications used for the chronic conditions depression, osteoporosis, and CVD, respectively. Antidepressants aim to correct chemical imbalances of neurotransmitters in the brain responsible for changes in mood and behaviour, bisphosphonates are used to prevent loss of bone density, and statins are used to lower cholesterol. While the minimum recommended length of antidepressant and bisphosphonate therapy is six months [24, 28] and three to five years [29], respectively, discontinuation of statins is generally not recommended [30].

Understanding how and to what extent non-MAP impacts health outcomes, leads to premature death, and exhausts valuable healthcare resources is important for improving clinical practice, developing health policies and prioritising research. Awareness of MAP patterns can help clinicians improve clinical practice and better manage health outcomes by regularly checking MAP, identifying reasons for non-MAP, and implementing interventions to improve MAP including better patient and clinician communication and shared decision making, better support from other health system stakeholders such as community care nurses, better medication packaging, and patient education. More accurate and informative MAP measures enable healthcare policymakers to better evaluate costs and benefits of MAP policies and interventions. In addition, better understanding of the link between MAP and HRUHC provides insights into prioritisation of future research.

This systematic literature review and meta-analysis aims to provide a comprehensive summary of the impact of non-MAP to three medication classes, antidepressants, bisphosphonates and statins, on HRUHC as measured by hospitalisation, emergency department (ED) presentation, visit to other healthcare service providers, healthcare cost and pharmacy cost. Evaluation of the three different medication classes under one systematic literature review permits understanding of whether different medication classes used with different patterns have different impacts on HRUHC using the same evaluation criteria.

## Methods

This review was conducted in accordance with a protocol (see dx.doi.org/10.17504/protocols.io.b4m4qu8w) developed using the process recommended by the Centre for Reviews and Dissemination [31] and written in accordance with the Preferred Reporting Items for Systematic Reviews and Meta-Analyses (PRISMA) guideline [32]. The abstraction and analysis of data were conducted by the first author based on the methods developed by all authors, and reviewed by all authors.

### Selection criteria

The scope of the systematic review was studies assessing the impact of non-MAP on HRUHC in antidepressants, bisphosphonates or statins. There was no restriction on the definition of MAP while HRUHC for the review included use of hospital, visit to healthcare service providers other than hospital, and healthcare cost components including medical cost and pharmacy cost. Studies were required to address the distinct impact of MAP to one of the three classes of medications on HRUHC, to be peer-reviewed, to be available as full articles, to be written in English, and to include quantitative analysis of the impact. The eligibility criteria are summarised in Table 1.

**Table 1:** Selection criteria.

Language	English	
**Publication**	Peer-reviewed, full articles	
**Type of study**		Review, correspondence (i.e., letters), editorial, expert opinion, discussion or commentary
**Year**	From November 2004 to April 2021	
**Method**	Quantitative analysis showing direct and clear impact of MAP on HRUHC ^a^	
**Exposure measure**	MAP to antidepressants, statins or bisphosphonates (either as a whole class of medication or as any individual medication from each class)	Combined MAP to multiple medications including a medication of interest
**Outcome measure**	Use of hospital; visits to other healthcare service providers; or healthcare cost components (e.g., medical cost, pharmacy cost)	

HRUHC = healthcare resource utilisation and healthcare cost; and MAP = medication adherence or persistence

a. The term, “direct and clear impact” is used to highlight that we exclude studies in which the impact of MAP on HRUHC can be implied from an analysis that does not measure the impact. For example, we exclude a study that describes MAP characteristics of a treatment group and measures the impact of the treatment on HRUHC.

### Search strategy

We used five search engines or registries: PubMed, Cochrane Library, ClinicalTrials.gov, JSTOR and EconLit. The period over which the search was conducted was 1 November 2004 to 30 April 2021. The period was set to achieve a balance between the number of studies included and focusing on a more recent period to ensure relevance of the findings. The search strategy was developed based on preliminary reviews of literature aiming at comprehensively including internationally used terminologies. Subsequent reviews of reference lists were conducted using the snowballing technique [33] to identify additional publications. The search strategy used with PubMed and Cochrane Library is displayed by item 38 in Table 2. For ClinicalTrials.gov, JSTOR and EconLit, due to the restriction on the length or form of search terms, we broadened our search specification to find studies having keywords (in their abstract for JSTOR and EconLit) showing the names of medications including statin, antidepressant, bisphosphonate or disphosphonate, and MAP or non-MAP using several words including adherence, compliance, nonadherence, noncompliance or persistence.

**Table 2:** Search strategy.

1. Medication Adherence (as MeSH Terms)
2. Patient Compliance (as MeSH Terms)
3. non-adherence (in either title or abstract)
4. Drug Therapy (as MeSH Terms)
5. medication (in either title or abstract)
6. (2 OR 3) AND (4 OR 5)
7. 1 OR 6
8. hmg coa statins (as MeSH Terms)
9. antidepressants (as MeSH Terms)
10. bisphosphonates (as MeSH Terms)
11. 8 OR 9 OR 10
12. Hospitalizations (as MeSH Terms)
13. hospital* (in either title or abstract)
14. Emergency Departments (as MeSH Terms)
15. emergency (in either title or abstract)
16. Practice, General (as MeSH Terms)
17. general practice* (in either title or abstract)
18. gp (in either title or abstract)
19. primary care (in either title or abstract)
20. visit* (in either title or abstract)
21. Costs and Cost Analysis (as MeSH Terms)
22. cost (in either title or abstract)
23. costs (in either title or abstract)
24. burden* (in either title or abstract)
25. accident and emergency (in either title or abstract)
26. A&E (in either title or abstract)
27. emergencies (in either title or abstract)
28. urgent medical aid service (in either title or abstract)
29.casualty department* (in either title or abstract)
30. secondary care (in either title or abstract)
31. specialist* (in either title or abstract)
32. outpatient* (in either title or abstract)
33. day patient* (in either title or abstract)
34. medical consultation* (in either title or abstract)
35. resource use* (in either title or abstract)
36. physician* (in either title or abstract)
37. 12 OR 13 OR …… OR 36
38. 7 AND 11 AND 37

MeSH = Medical Subject Headings

Note: Asterisks were used to include plurals.

After removing duplicates, abstracts were screened to arrive at the eligible or possibly eligible studies for a full-text review. The full-text review was conducted to exclude articles not meeting the eligibility criteria, and to extract data.

### Data extraction

Extracted information for the review includes authors, country, year of publication, study type (e.g., retrospective cohort study), medications studied, data period, characteristics of cohort, statistical method of analysis, measure of MAP, reported MAP of cohort, and summary of impact of non-MAP on HRUHC. In cases where both adjusted estimates (incorporating covariates) and unadjusted estimates (not incorporating covariates) for the relationship between MAP and HRUHC were reported, we summarise the adjusted estimates only because the inclusion of covariates facilitates meaningful interpretation and comparison. We report statistically significant or insignificant coefficients showing the relationships between MAP and HRUHC rather than absolute amounts of change in HRUHC where possible. This is to account for different baseline levels of HRUHC and different contexts (e.g., time frame, country). Other supporting information (e.g., covariates, reported conflicts of interest) was also collected to support quality assessments.

### Quality criteria

Quality of study and risk of bias were evaluated using the Quality Assessment Tool for Observational Cohort and Cross-Sectional Studies [34]. This tool is used to assess the quality of observational cohort or cross-sectional studies [e.g., 35, 36] and was considered appropriate for the review because all included studies were observational cohort studies. The tool rates a study quality as good, fair or poor based on 14 questions about study objectives, sample selection, definition and use of exposure and outcome, analysis method and risk of bias.

### Aggregate data meta-analysis

A meta-analysis was conducted for a certain relationship between MAP and HRUHC when reported for at least two different cohorts of population. Note that “different cohorts” here refers to both cohorts in different studies and different cohorts within a single study. For multiple results to be synthesised, the measure of MAP (e.g., adherence defined by amount of medication used relative to total amount recommended greater than 80% over six months) was required to be comparable in terms of the length of measuring period and which aspect of MAP is measured (e.g., adherence, adherence rate, persistence). In addition, medication class (e.g., antidepressants) and type of HRUHC (e.g., hospitalisation cost) were required to be the same. When the requirements were met, the combined result was estimated using the inverse-variance method which allocates each study a weight equal to the inverse of the variance of the effect estimate [37], i.e.

$$inverse-variance\;weighted\;average=\frac{\sum Y_{i}(1/{SE}_{i}^{2})}{\sum \left(1/{SE}_{i}^{2}\right)}$$

where *Y*_*i*_ is the effect estimated in the *i*^th^ study, SE_*i*_ is the standard error of the estimate, and the summation is across all studies. When the standard error was not reported, it was approximated from the confidence interval or p-value [38]. Analysis was implemented using RevMan v5.4 [39].

## Results

### Study selection

**Fig 1:**
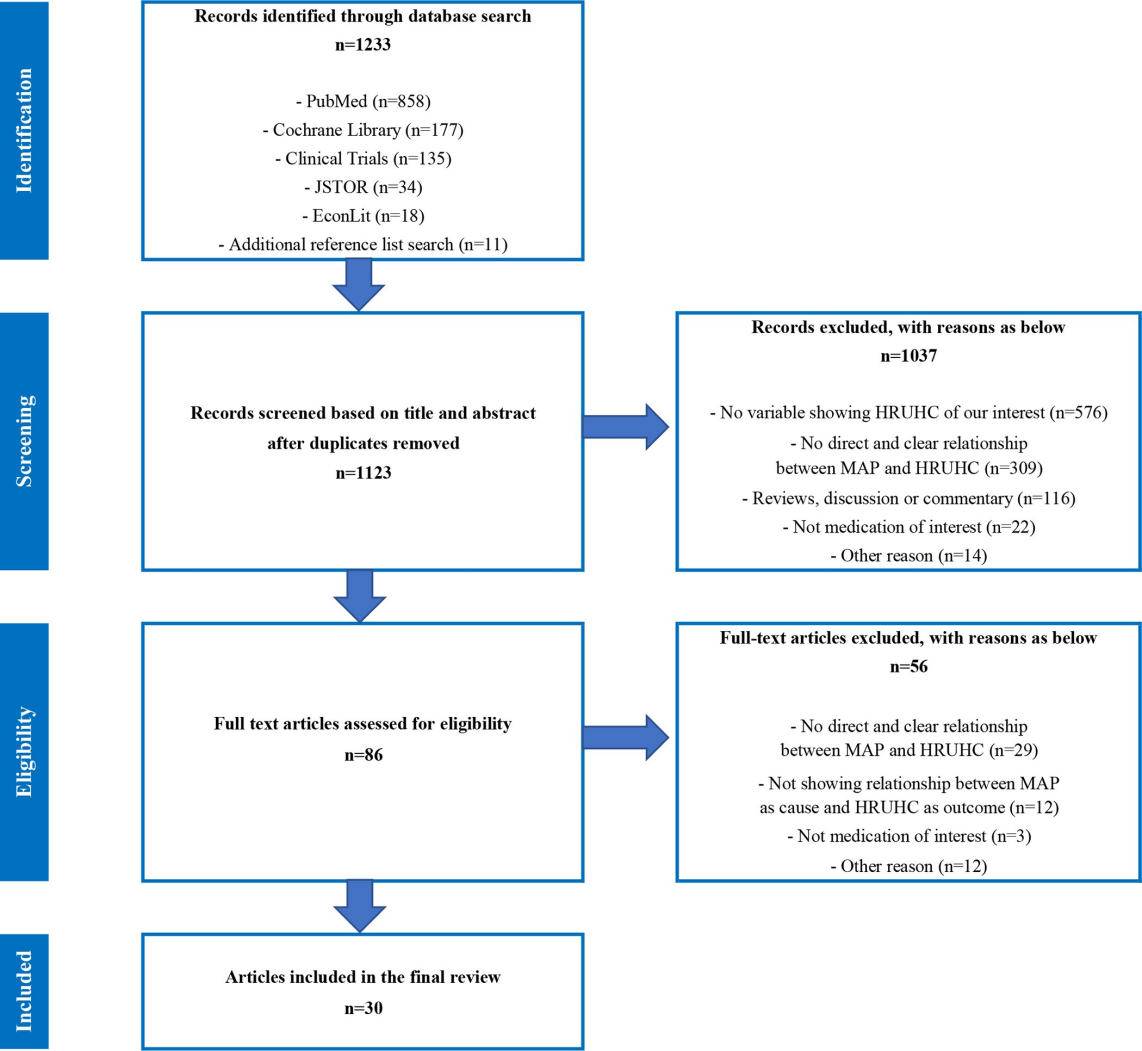
Preferred Reporting Items for Systematic Reviews and Meta-Analyses (PRISMA) flow diagram. The PRISMA diagram details the search and selection process applied during the overview.

As shown by the PRISMA flow diagram in Fig 1, the initial search, abstract screening and full-text review have left 30 articles for the review. The characteristics of individual studies on antidepressants, bisphosphonates and statins are summarised in Tables 3–5, respectively. Study findings of the impacts of MAP to antidepressants, bisphosphonates and statins on several different types of HRUHC are summarised in Tables 6–8, respectively. A summary of the directions of the impact of MAP on HRUHC based on the study findings is presented in Table 9.

**Table 3:** Characteristics of reviewed studies on antidepressants.

Paper	Country	Study type	Medication	Data period and breakdown of the period	Size, age and gender of cohort	Description of cohort
Eaddy et al. (2005) [46]	US	Retrospective cross-sectional using health research/ claims database	All SSRI medications including citalopram, fluoxetine, paroxetine immediate-release, paroxetine controlled-release, and sertraline	Data period: Jan 2001—Jun 2003.	56,753	Patients diagnosed with depression and newly prescribed SSRIs
				Index period: Jun 2001—Jun 2002		
				Baseline period: 6 months to index		
				MAP and follow-up period: 12 months from index	18+, All	
Katon et al. (2005) [49]	US	Retrospective cross-sectional using health research/ claims database	Bupropion hydrochloride, bupropion hydrochloride sustained-release, citalopram hydrobromide, escitalopram oxalate, fluoxetine hydrochloride, mirtazapine, nefazodone hydrochloride, paroxetine hydrochloride, paroxetine hydrochloride controlled release, sertraline hydrochloride, venlafaxine hydrochloride, and venlafaxine hydrochloride extended-release	Data period: 2001–2003	8,040	Coronary artery disease, dyslipidaemia or diabetes patients newly prescribed antidepressants
				Index period: Jul 2001—Dec 2002		
				Baseline period: 6 months to index		
				MAP period: 6 months from index		
				Follow-up period: 12 months from index		
Cantrell et al. (2006) [40]	US	Retrospective cohort using health research/ claims database	SSRIs including fluoxetine, sertraline, citalopram, escitalopram, paroxetine immediate-release, and paroxetine controlled-release	Data period: Jan 2001—Jun 2003	22,947	Patients diagnosed with depression and newly prescribed SSRIs
				Index period: Jul 2001—Jun 2002		
				Baseline period: 6 months to index		
				MAP period: 6 months from index	18+, All	
				Follow-up period: 1 year from index		
Robinson et al. (2006) [43]	US	Retrospective cohort using health research/ claims database	All classes of antidepressants	Data period: Jan 2001—Sep 2004	60,386	Patients newly diagnosed with depression and prescribed antidepressants
				Follow-up period: 6 months from index	18+, All	
Stein et al. (2006) [68]	US	Retrospective cohort using health research/ claims database	Venlafaxine, venlafaxine extended-release, fluoxetine, sertraline, paroxetine, paroxetine controlled-release, citalopram, escitalopram, and fluvoxamine	Data period: 2001–2003	13,085	Patients prescribed antidepressants and newly diagnosed with anxiety or anxiety and comorbid depression
				Index period: Jul 2001—Dec 2002		
				Baseline period: 6 months to index	18+, All	
				MAP and follow-up period: 12 months from index		
Tournier et al. (2009) [48]	Canada	Retrospective cohort using health research/ claims database	Citalopram, fluoxetine, fluvoxamine, paroxetine, sertraline, nefazodone, trazodone, and venlafaxine	Data period: 1999–2001	12,825	Patients newly prescribed antidepressants
				Index period: 2000		
				Baseline period: 1 year to index		
				MAP period: 180 days from index	66+, All	
				Follow-up period: 1 year from index		
Ereshefsky et al. (2010) [66]	US	Retrospective cohort using health research/ claims database	Citalopram, escitalopram, fluoxetine, paroxetine, and sertraline	Data period: Jan 2002 –Jun 2006	45,481	Patients newly diagnosed with depression and prescribed antidepressants
				Index period: 2003–2004		
				Baseline period: 12 months to index		
				MAP period: 6 months to index	18+, All	
				Follow-up period: 18 months from index		
Albrecht et al. (2017) [45]	US	Retrospective cohort using health research/ claims database	All classes of antidepressants	Data period: 2006–2012	16,075	Patients diagnosed with depression and chronic obstructive pulmonary disease
					All, All	
Vega et al. (2017) [44]	US	Retrospective cohort using health research/ claims database	All classes of antidepressants	Data period: Jan 2012—Jun 2014	1,361	Type-2 diabetes patients newly diagnosed with major depression
				Index period: Jul 2012—Jun 2013		
				Baseline period: 6 months to index	18+, All	
				MAP period: 180 days from index		
				Follow-up period: 12 months from end of MAP period		
Aznar-Lou et al. (2018) [47]	Spain	Longitudinal retrospective cohort study using health research/ claims database	All SSRI medications	Data period: 2011–2014	79,642	Patients newly prescribed SSRI and diagnosed with depressive disorder
					16–65, All	

SSRI = selective serotonin reuptake inhibitor

**Table 4:** Characteristics of reviewed studies on bisphosphonates.

Paper	Country	Study type	Medication	Data period and breakdown of the period	Sample size Age and gender of cohort	Description of cohort
Briesacher et al. (2007) [50]	US	Retrospective cohort using health research/ claims database	Alendronate, and risedronate	Data period: 2000–2004	17,988	Patients diagnosed with osteoporosis and newly prescribed bisphosphonates
				Baseline period: 1 year to index		
				MAP and follow-up period: 31 Dec 2004 from index	40+, All	
Sunyecz et al. (2008) [41]	US	Retrospective cohort using health research/ claims database	Alendronate, and risedronate	Data period: Jan 1999—Jun 2005	32,944	Patients newly prescribed bisphosphonates
				Index period: Jan 2000—Jun 2002		
				Baseline period: 1 year to index	45+, Female	
				MAP and follow-up period: 3 or more years from index		
Eisenberg et al. (2015) [52]	US	Retrospective cohort using health research/ claims database	Alendronate, risedronate, and ibandronate	Data period: Jan 2006—Sep 2012	27,905	Patients diagnosed with osteoporosis and prescribed bisphosphonates
				Index period: Jan 2007—Sep 2010		
				Baseline period: 1 year to index	55+, All	
				MAP period: 1 year from index		
				Follow-up period: 1 year from end of MAP period		
LaFleur et al. (2015) [70]	US	Retrospective cohort using health research/ claims database	Oral alendronate, oral or injectable ibandronate, oral risedronate, and injectable zoledronic acid	Data period: Jan 2003—Dec 2011	35,650	Veterans newly or continually prescribed bisphosphonates
					50+, Female	
Ferguson et al. (2016) [69]	UK	Retrospective cohort using health research/ claims database	Alendronate, risedronate, ibandronate, and etidronate	Data period: 1999–2008	36,320	Patients diagnosed with postmenopausal osteoporosis and newly prescribed bisphosphonates
				Index period: 2000–2007	50+, Female	
Kjellberg et al. (2016) [53]	Denmark	Retrospective cohort using health research/ claims database	Alendronate, risedronate, and ibandronate	Data period: 2002–2010	38,234	Patients newly prescribed bisphosphonates
				Index period: 2003–2008		
				Baseline period: 1 year to index		
				MAP period: 1 year from index	55+, Female	
				Follow-up period: 1 year from end of MAP period		
Sharman Moser et al. (2016) [51]	Israel	Retrospective cohort using health research/ claims database	Alendronate, and risedronate	Data period: 2004–2013	17,770	Patients newly prescribed bisphosphonates
				Index period: 2005–2011		
				Baseline period: 1 year to index		
				MAP period: 1 year from index	55+, Female	
				Follow-up period: 1 year from end of MAP period		

**Table 5:** Characteristics of reviewed studies on statins.

Paper	Country	Study type	Medication	Data period and breakdown of the period	Size, age and gender of cohort	Description of cohort
Cheng et al. (2006) [54]	Hong Kong	Prospective observational cohort study	Simvastatin and atorvastatin	Index period: Jan 2003—Jun 2003	83	Coronary heart disease patients prescribed statins for less than 12 months
				MAP and follow-up period: Jul 2003—Dec 2003	18+, All	
Gibson et al. (2006) [56]	US	Retrospective cohort using health research/ claims database	All classes of statins	Data period: 2000–2003	117,366	New or incident, continuing or prevalent statin users
				MAP period: Jul 2001—Dec 2002		
				Follow-up period: 2003	18+, All	
Stuart et al. (2009) [42]	US	Retrospective cohort using health research/ claims database and survey data	All classes of statins, oral anti-diabetes agents and Angiotensin converting enzyme inhibitors	Data period: 1997–2004	7,441 (4,641)^a^	Diabetes patients
					All, All	
Aubert et al. (2010) [57]	US	Retrospective cohort using health research/ claims database	All classes of statins	Data period: Jan 2000—Jun 2004	10,227	Patient newly prescribed statins
				Index period: Jul 2001—Jun 2002		
				Baseline period: 6 months to index		
				MAP period: 2 years from index	18+, All	
				Outcome period: 1 year from end of MAP period		
Pittman et al. (2011) [61]	US	Retrospective cohort using health research/ claims database	Atorvastatin, fluvastatin, lovastatin, pravastatin, rosuvastatin, simvastatin, and simvastatin/ezetimibe	Data period: Jan 2007—Jun 2009	381,422	Statin users not including those newly prescribed statins
				Index period: Jan 2008—Jun 2008		
				Baseline period: Jan 2007—Dec 2007	18–61, All	
				MAP period: 1 year to index		
				Follow-up period: Jan 2008—Jun 2009		
Stuart et al. (2011) [63]	US	Longitudinal study using survey data	Statins and renin–angiotensin–aldosterone system inhibitors	Data period: 1997–2005	3,765 (1,139) ^b^	Diabetes patients using studied medications
				Follow-up period: until participants completed their survey tenure, were lost to follow-up, were admitted to long-term care facility, or died	All, All	
Wu et al. (2011) [58]	US	Retrospective cohort using health research/ claims database	All classes of statins	Data period: 2004–2006	1,705	Diabetes patients newly prescribed statins
				Index period: 2005	18+, All	
				Baseline period: 1 year to index		
				MAP and follow-up period: 1 year from index		
Chen et al. (2012) [59]	US	Retrospective cohort using health research/ claims database	All classes of statins	Data period: Dec 2009—Dec 2010	30,139	Patients discharged from the acute inpatient setting with diabetes
				Index period: Dec 2009—Nov 2010		
				Follow-up period: 30 days from end of MAP period	19+, All	
Roberts et al. (2014) [64]	US	Retrospective cohort using health research/ claims database	All classes of statins, sulfonylureas, thiazolidinediones, metformin, angiotensin converting enzyme inhibitors, angiotensin II, receptor blockers, calcium channel blockers, or diuretics	Data period: 2009–2011	7,180	Users of studied medications
				MAP and follow-up period: 6 months from index	(1,349) ^b^	
					All, All	
Zhao et al. (2014) [60]	US	Retrospective cohort using health research/ claims database	All classes of statins	Data period: 2008–2011	10,312	Patients newly prescribed statins
				Index period: 2009		
				Baseline period: 1 year to index	18–64, All	
				MAP and follow-up period: 1 year from index		
Li and Huang (2015) [62]	Taiwan	Retrospective cohort using health research/ claims database	Lovastatin, pravastatin, simvastatin, fluvastatin, and atorvastatin	Data period: 2001–2007	19,371	Patients newly prescribed statins
				MAP and follow-up period: 3 years (or until hospitalisation) from index	45+, All	
Mehta et al. (2019) [55]	US	Retrospective cohort secondary analysis using randomised clinical trial	All classes of statins, aspirins, beta-blockers, antiplatelet agents	Index period: Mar 2013—Jan 2015	1,000	Patients prescribed at least 2 of 4 study medications, hospitalised and discharged to home with diagnosis of acute myocardial infarction
				MAP period: 12 months from index	(682) ^b^	
				Follow-up period: Up to 60 days from end of MAP period	18–80, All	
Kirsch et al. (2020) [65]	Germany	Retrospective cohort using health research/ claims database	All classes of statins, beta-blockers, antiplatelet agents and angiotensin converting enzyme inhibitors	Data period: 2008–2014	3,627	Patients with at least 1 hospitalisation with acute myocardial infarction
				Patient selection period: 2009–2011		
				MAP and follow-up period: 2012–2014	(Not provided) ^b^	
					All, All	

a. The medications include non-statin medications included in each study, if any, for information. However, the review extracted the findings related to statins only.

b. Number of statin users

**Table 6:** Impact of adherence or persistence to antidepressants on healthcare resource utilisations and healthcare costs.

Paper	Method of analysis	Measure of MAP	Reported MAP of cohort	Impact of MAP on HRUHC
				(95% CI is in square brackets)
Eaddy et al. (2005) [46]	Analysis of covariance	1-year adherence is categorised into five mutually exclusive groups:	36%, 16%, 13%, 12% and 23% were in <90 days, ≥90 days, Partial, Titration and Change category, respectively.	(Annual average per-patient provider submitted charge for <90 Days, ≥ 90 Days, Partial, Titration and Change with statistical significance reported as compared to ≥90-day category)
		1. <90 days: not having at least 90 days of continuous therapy without 15-day gap.		Inpatient: $2,094 (p>0.05), $1,446, $2,040 (p>0.05), $1,996 (p>0.05) and $2,386 (p>0.05), respectively.
				Outpatient: $1,427 (p>0.05), $1,302, $1,319 (p>0.05), $1,499 (p>0.05) and
		2. ≥90 days: having 90 days or more of continuous therapy without 15-day gap, titration in dose and evidence of receiving another antidepressant.		$1,868 (p>0.05), respectively.
				Emergency department: $309 (p>0.05), $159, $177 (p>0.05), $238
				(p>0.05) and $302 (p>0.05), respectively.
				Physician: $1,434 (p>0.05), $1,290, $1,334 (p>0.05), $1,584 (p>0.05) and $2,007 (p>0.05), respectively.
		3. Partial: having at least 90 days of continuous therapy with at least one 15-day gap after 90 days and without titration in dose and evidence of receiving another antidepressant.		All medical charges: $6,289 (p<0.001), $5,143, $5,909 (p<0.05), $6,375 (p<0.001) and $7,858 (p<0.001), respectively.
				SSRI charges: $508 (p>0.05), $886, $802 (p>0.05), $1,066 (p>0.05) and $1,172 (p>0.05), respectively.
		4. Titration: having at least 90 days of continuous therapy with an increase in dosage and without 15-day gap and evidence of receiving another depressant.		Medical + SSRI charges: $6,797 (p<0.001), $6,029, $6,711 (p<0.001), $7,441 (p<0.001) and $9,030 (p<0.001), respectively.
				Other pharmacy charges: $1,032 (p>0.05), $1,424, $1,236 (p>0.05), $1,503 (p>0.05) and $1,939 (p>0.05), respectively.
		5. Change: having at least 90 days of continuous therapy with evidence of receiving another antidepressant and without 15-day gap.		Total charges: $7,829 (p>0.05), $7,453, $7,947 (p<0.05), $8,944 (p<0.05) and $10,969 (p<0.05), respectively.
Katon et al. (2005) [49]	Multivariate log-linear regression	Adherence is defined as 6-month MPR ≥ 80% and absence of 15-day gap in first 90 days.	38% were adherent.	(Reporting figures for adherent vs non-adherent patients)
				Less inpatient charges for coronary artery disease (CAD)/dyslipidaemia, diabetes and CAD/dyslipidaemia and diabetes patients for -14% [-0.29, 0.01] at p = 0.08, -45% [-0.72, -0.19] at p = 0.001 and -23% [-0.69, 0.23] at p = 0.33, respectively.
				Less outpatient charges for CAD/dyslipidaemia, diabetes and CAD/dyslipidaemia and diabetes patients for -6% [-0.12, 0.002] at p = 0.06, -10% [-0.21, 0.02] at p = 0.09 and -22% [-0.40, -0.05] at p = 0.01, respectively.
				Less total medical charges for CAD/dyslipidaemia, diabetes and CAD/dyslipidaemia and diabetes patients for -6% [-0.13, -0.001] at p = 0.05,
				-12% [-0.23, -0.005] at p = 0.04 and -20% [-0.38, -0.02] at p = 0.03, respectively.
Cantrell et al. (2006) [40]	Analysis of covariance	Three methods to measure 180-day MAP are compared:	MPR method: 43% were adherent.	Lower total cost excluding antidepressant prescription costs ($2,924 vs $3,435, $3,012 vs $3,435 and $3,006 vs $3,440 for adherent vs nonadherent patients using LOT, MPR and MPR/LOT methods, respectively with p<0.0001).
		1. MPR method: adherence is defined as 180-day MPR ≥ 80%.	LOT method: 45% were adherent.	
		2. Length of Therapy (LOT) method: adherence is defined as no 15-day gap in 180 days.	MPR/LOT method: 43% were adherent.	
		3. MPR/LOT method: adherence is defined as 180-day MPR ≥ 80% and no 15-day gap in at least 90 days in 180 days.		
Robinson et al. (2006) [43]	Multivariate exponential conditional mean regression	A patient is adherent when:	19% were adherent.	Extra total healthcare cost of $806 (p<0.001) for adherent patients.
		(1) at least 84 days’ supply during first 114 days;		Extra mental-health specific healthcare cost of $644 (p<0.001) for adherent patients.
		(2) at least 180 days’ supply during first 214 days; and		Additional information ^a^
				Median unadjusted total healthcare cost: $5,169 (adherent patients) $2,734 (nonadherent patients).
		(3) at least three contacts with healthcare providers including at least one contact with a practitioner licensed to prescribe.		Median unadjusted mental health cost: $1,922 (adherent patients) vs. $677 (nonadherent patients).
Stein et al. (2006) [68]	Inferential analyses, analysis of covariance	Adherence is defined as 1-year MPR ≥ 80% and patients are categorised into four groups including: nonadherent; adherent, no change; adherent, dosage was titrated; and adherent, change in medication.	15% were adherent.	(Reporting figures for adherent vs non-adherent patients)
			57% were non-adherent.	1. Patients with anxiety disorders alone
				Lower medical care cost at $2,640 (SD 5,341) vs $3,070 (SD 5,932), at p<0.05.
			19% were adherent, dosage titrated.	Greater anxiety medication cost at $700 (SD 261) vs $277 (SD 259), at p<0.05.
			10% were adherent, change in medication.	Greater other medication cost at $909 (SD 2,129) vs $771 (SD 1,329), at p>0.05.
				Greater total cost at $4,248 (SD 6,001) vs $4,119 (SD 5,366), at p>0.05.
				2. Patients with anxiety and depressive disorders
				Lower medical care cost at $3,220 (SD 5,323) vs $3,807 (SD 5,932), at p>0.05.
				Greater anxiety medication cost at $691 (SD 272) vs $338 (SD 281), at p<0.05.
				Greater other medication cost at $892 (SD 2,243) vs $801 (SD 1,358), at p>0.05.
				Greater total cost at $4,803 (SD 6,942) vs $4,946 (SD 6,394), at p>0.05.
Tournier et al. (2009) [48]	Multivariate logistic regression model	Non-persistence is defined as a treatment duration of less than 180 days without a 30-day gap.	Percentages of non-persistent treatment by antidepressant class	(Reporting figures for persistent vs non-persistent patients)
				Greater cost of initial antidepressants ($321 [316, 326] vs $102 [98, 107]).
			1. SSRI: 53%	Greater cost of other medications ($1,444 [1,417, 1,472] vs $1,193 [1,163, 1,224]
			2. Serotonin noradrenergic reuptake inhibitors: 53%	Greater cost general practice services ($371 [363, 380] vs $355 [346, 365]).
				Lower cost of psychiatric visits ($37 [31, 42] vs $42 [38, 47]).
				Lower cost of other specialty visits ($420 [404, 436] vs $462 [444, 480]).
			3. Others: 65%	Lower non-psychiatric hospitalisation costs ($1,768 [1,632, 1,908] vs $2,200 [2,44, 2,356]).
				No significant difference in psychiatric hospitalisation costs ($64 [44, 80] vs $52 [32, 72]).
Ereshefsky et al. (2010) [66]	Multivariate GLM with gamma distribution and log link	Persistence is defined as treatment gap not greater than 30 days during 180 days.	19% were persistent.	Higher healthcare costs in non-persistent patients (RR 1.054 [0.999; 1.112], p = 0.055).
Albrecht et al. (2017) [45]	GLM with binomial distribution and complementary log-log link	Rolling 3-month average from 30-day PDCs, then categorised using two methods:	55% achieved average PDC ≥ 80%.	(Reporting figures for the second categorisation of MAP)
		1. 0%, <20%, ≥20% and <40%, ≥40% and <60%, ≥60% and <80%, and ≥80%.		Lower ED visits (HR 0.74 [0.70, 0.78], insignificant) and all-cause hospitalisations (HR 0.77 [0.73, 0.81], insignificant) for adherence ≥80%, compared to adherence = 0%.
		2. 0%, >0% and <80%, and ≥80%.		Lower ED visits (HR 0.72 [0.68, 0.76], insignificant) and all-cause hospitalisations (HR 0.77 [0.72, 0.82], insignificant) for adherence >0% and <80%, compared to adherence = 0%.
Vega et al. (2017) [44]	Multivariate GLM with gamma distribution and log link with bootstrapping	1. Adherence is defined as 180-day MPR ≥ 80%.	36% were adherent.	(Compared to nonadherent, nonpersistent and those who are not adherent/persistent)
		2. Persistence is defined as absence of a 15-day gap in 180 days.	32% were persistent.	Marginal total cost of -$350 [-$462, $-247], $493 [$473, $513] and -$1,165 [-$1,280, -$1,060] for adherent, persistent and adherent/persistent patients, respectively.
		3. Adherence and persistence is defined as 180-day MPR 80% and absence of a 15-gap in first 90 days.	31% were adherent and persistent.	Marginal medical cost of -$2,290 [-$2,430, -$2,162], -$183 [-$195, -$173] and -$2,152 [-$2,283, -$2,031] for adherent, persistent and adherent/persistent patients, respectively.
				Marginal pharmacy cost of $1,940 [$1,870, $2,007], $676 [$652, $700] and $987 [$952, $1,021] for adherent, persistent and adherent/persistent patients, respectively.
				Additional information ^a^
				For all patients combined, mean total cost, medical cost and pharmacy cost was $21,112, $15,697 and $5,416, respectively.
Aznar-Lou et al. (2018) [47]	Multivariate logistic regression	Initial non-adherence is defined as not filling prescription for newly prescribed SSRI in the month of prescription or the following month.	15% were initially non-adherent.	Less general practice visits (OR of 0.82 [0.79, 0.84], p<0.05) for initially non-adherent patients.
				No significant difference in specialist visits (OR of 1.04 [0.99, 1.08], p>0.05).

a. Additional information is provided for comparison purpose when the reported figures are not in relative terms.

CR = cost ratio; ED = emergency department; GLM = generalized linear model; HR = hazard ratio; HRUHC = healthcare resource utilisations and healthcare costs; MAP = medication adherence or persistence; MPR = medication possession ratio; OR = odd ratio; PDC = proportion of days covered; RR = relative risk; SD = standard deviation; SE = standard error; and SSRI = selective serotonin reuptake inhibitor

**Table 7:** Impact of adherence or persistence to bisphosphonates on healthcare resource utilisations and healthcare costs.

Paper	Method of analysis	Measure of MAP	Reported MAP of cohort	Impact of MAP on HRUHC
				(95% CI is in square brackets)
Briesacher et al. (2007) [50]	Multivariate regression	Five categories of each follow-up year’s MPR, 0–19%, 20–39%, 40–59%, 60–79%, and 80–100%.	(For the five MAP categories)	(Compared to MPR of 0–19%, below findings significant at p<0.1)
			Year 1: 43%, 13%, 10%, 14%, and 20%.	Marginal total costs are -$859, -$474, -$366 and $151 for MPR 80–100%, 60–79%, 40–59%, and 20–39%, respectively.
			Year 2: 35%, 11%, 8%, 8%, and 39%.	Marginal prescription costs are $997, $923, $402 and $160 for MPR 80–100%, 60–79%, 40–59%, and 20–39%, respectively.
			Year 3: 31%, 10%, 7%, 8%, and 44%.	Marginal costs of hospitalisation are -$3233, -856, -$6221, -$585 for MPR 80–100%, 60–79%, 40–59%, and 20–39%, respectively.
				Marginal outpatient costs are -$445, -$538, -$236, $60 for MPR 80–100%, 60–79%, 40–59%, and 20–39%, respectively.
				Additional information ^a^
				Full costs (e.g., total outpatient cost) were not reported.
Sunyecz et al. (2008) [41]	GLM and logistic multivariate regression	1. Persistence is defined as no gap ≥ 30 days for follow-up period.	21% were persistent.	8.9% [-0.122, -0.056] at p<0.001 and 3.5% [-0.064, -0.007] at p = 0.014 lower total cost for persistent and compliant patients, respectively.
		2. Compliance is defined as 3-year MPR ≥ 0.80.	37% were compliant.	Almost 50% lower risk of hospitalisation and 1.6 times greater likelihood of outpatient visits for persistent patients.
Eisenberg et al. (2015) [52]	GLM with gamma distribution and log link	Adherence is defined as 1-year MPR ≥ 70%.	41% were adherent.	(Reporting for adherent vs non-adherent patients)
				9% (SE 1.04 at p = 0.007) lower osteoporosis-related costs.
				3% (SE 1.03 at p = 0.298) lower total costs insignificantly.
LaFleur et al. (2015) [70]	Generalized estimating equations (GEE) with gamma distribution and log link	Longitudinal quarterly MAP (starting from the first prescription filled at least 6 months after the first outpatient encounter) is categorised into four types:	(Not mutually exclusive)	(Compared to non-switchers)
			19% were non-switching.	14% [0.06, 0.21], 5% [0.03, 0.08] and 17% [0.13, 0.20] greater total cost for switchers, discontinuers and reinitiators, respectively.
			1% were switching.	
			80% were discontinuing.	14% [-0.29, 0], 106% [-1.14, 0.98] and 22% [-0.32, -0.11] less osteoporosis-related cost for switchers, discontinuers and reinitiators, respectively.
		1. Non-switching: continuing on index bisphosphonate.		
			4% were reinitiating.	
		2. Switching: switching from index bisphosphonate to a different bisphosphonate.		
				66% [-0.78, -0.54], 234% [-2.37, -2.31] and 58% [-0.61, -0.56] less osteoporosis-related pharmacy cost for switchers, discontinuers and reinitiators, respectively.
		3. Discontinuing: presence of gap ≥ 90 days.		
		4. Reinitiating: restarting index bisphosphonate after discontinuation or switch.		
Ferguson et al. (2016) [69]	Multivariate generalized linear mixed model	Discontinuation is defined as a gap greater than 3 months.	26%, 20%, 16% and 38% were persistent for 0–12 months, 12–24 months, 24–36 months and 36 months or more, respectively.	Greater HRUHC for persistence of 0–12 months as much as HR 2.14 [1.38, 3.33] at p = 0.0007, HR 1.98 [1.63, 2.41] at p<0.0001 and HR 1.29 [1.16, 1.44] at p<0.0001, compared to persistence of 12–24 months, 24–36 months and 36 months or more, respectively.
		Persistence, defined as the duration of use of oral bisphosphonates, is categorised into four groups, 0–12 months, 12–24 months, 24–36 months and 36 months or more.		
			85.1% achieved MPR of 80% or more.	Greater HRUHC for persistence of 12–24 months with HR 5.29 [1.94, 14.4] at p = 0.0012 compared to persistence of 24–36 months.
				No significant differences between other groups (p>0.5).
		MPR is calculated for the period before discontinuation.		
Kjellberg et al. (2016) [53]	GLM with poisson distribution and log link and with gamma distribution and log link	Compliance is defined as 12-month MPR≥70%.	70% were compliant.	(Reporting figures for non-compliant vs compliant patients, all at p<0.001, SE in square brackets)
				Osteoporosis-related resource use: inpatient admissions (HR 1.32 [0.05]), outpatient services (HR 1.38 [0.03]), prescription claims (HR 0.45 [0.01]).
				All-cause resource use: inpatient admission (HR 1.31 [0.02]), emergency room visits (HR 1.34 [0.02]), outpatient services (HR 1.22 [0.01]), prescription claims (HR 0.90 [0.00]).
				Osteoporosis-related costs: medical only (CR 1.18 [0.01]), medical and prescription (CR 1.42 [0.01]).
				All-cause costs: medical only (CR 1.25 [0.01]), medical and prescription (CR 1.22 [0.01]).
Sharman Moser et al. (2016) [51]	GLM with gamma distribution and log link and poisson distribution	Adherence is defined as 12-months MPR ≥ 70%.	51.1% were adherent.	(Reporting figures for nonadherent vs adherent patients)
		Non-adherence includes discontinuation defined as a gap ≥ 60 days.		Greater all-cause healthcare cost (CR 1.027 [0.996, 1.059], p<0.090).
				For age group 75 or older, greater all-cause healthcare cost (CR 1.134 [1.048, 1.227], p = 0.002).
				For age group 55–64 and 65–74, no significant differences in all-cause healthcare cost (p>0.4).

a. Additional information is provided for comparison purpose when the reported figures are not in relative terms.

CR = cost ratio; ED = emergency department; GLM = generalized linear model; HR = hazard ratio; HRUHC = healthcare resource utilisations and healthcare costs; IRR = Incident rate ratio; MAP = medication adherence or persistence; MPR = medication possession ratio; OR = odd ratio; PDC = proportion of days covered; RR = relative risk; SD = standard deviation; and SE = standard error

**Table 8:** Impact of adherence or persistence to statins on healthcare resource utilisations and healthcare costs.

Paper	Method of analysis	Measure of MAP	Reported MAP of cohort	Impact of MAP on HRUHC
				(95% CI is in square brackets)
Cheng et al. (2006) [54]	Backward multiple regression analysis	Adherence was monitored with two follow-up visits scheduled at 3 and 6 months and also using the statin prescription dispensed in a bottle with the Medication Event Monitoring System.	Median dose-count adherence: 96.4%	No statistically significant relationship found at p<0.05 regarding total direct medical cost per member per month involving clinic visits, statin medications, laboratory tests on lipids and management of CHD events.
		Adherence was assessed by dose-count defined as the percentage of doses taken, and dose-time was defined as the percentage of doses taken within the suggested time interval.	Median dose-time adherence: 88.1%	
Gibson et al. (2006) [56]	Logit model and GLM with gamma distribution and log link	Adherence is defined as 18-month MPR ≥ 80%.	Mean MPR for new users: 28%	(Reporting figures for adherent vs non-adherent patients)
				New users
				Higher physician office visits (OR of 2.526 [SE 0.930], p<0.01) and lower CHD hospitalisations (OR of 0.414 [SE 0.203], p<0.1).
				No significant difference in ED visits, hospitalisations and all types of cost (p>0.1).
			Mean MPR for continuing users: 59%	Continuing users
				Lower ED visits (OR of 0.220 [SE 0.057], p<0.01), lower hospitalisation (OR of 0.0568 [SE 0.177], p<0.1) and lower CHD hospitalisations (OR of 0.18 [SE 0.09], p<0.01).
				No significant difference in physician office visits (p>0.1).
				Higher prescription drug spending (coefficient estimate of 0.703 [SE 0.069], p<0.1).
				No significant difference in other costs (p>0.1).
Stuart et al. (2009) [42]	GLM with gamma distribution and log link, poisson model and logistic regression model	Annual number of prescription fills per class per year	Not reported.	(With one additional prescription fill)
				0.5% [-0.9, -0.04] at p<0.05 lower hospitalization risk.
				0.05 [-0.09, -0.02] at p<0.01 fewer inpatient days.
				$107 [–193, –21] at p<0.05 less Medicare spending in 2006 USD.
Aubert et al. (2010) [57]	GLM and logistic regression model	Adherence is defined as 2-year MPR ≥ 80%.	34% were adherent.	(Reporting for adherent vs non-adherent patients)
				Lower percentage of patients hospitalized (16% vs 19%, p <0.01) and fewer hospitalizations (25 vs 33 per 100 patients, p <0.01).
				Lower total medical cost excluding cost of statin therapy ($4,040 [$3,601, $4,478] vs $4,908 [$4,594, $5,222], p <0.01).
				Lower total medical cost including cost of statin therapy ($4909 [$4470, $5347] vs $5290 [$4976, $5604], p<0.01).
Pittman et al. (2011) [61]	Logistic regression and GLM	Three categories of 365-day MPR, 80%-100%, 60%-79% and 0%-59%.	15.1%, 17.3% and 67.6% of patients achieved MPR of 0–59%, 60–79% and 80% or more, respectively.	(p-value is not applicable to MPR of 80% or more which is the reference category of analysis)
				Compared to MPR of 80% or more, greater cardiovascular hospitalisation for MPR of 0–59% (OR of 1.26 [1.21, 1.31] at p<0.05) and MPR of 60–79% (OR of 1.12 [1.08, 1.16] at p<0.05).
				All-cause total healthcare costs of $11,101 (SE 84.3, p<0.001), $10,609 (SE 77.7, p<0.001) and $10,198 (SE 39.4) for MPR of 0–59%, 60–79% and 80% or more, respectively.
				Cardiovascular medical costs of $2,689 (SE 43.9, p<0.001), $2,583 (SE 40.4, p<0.001) and $2,395 (SE 20.5) for MPR of 0–59%, 60–79% and 80% or more, respectively.
				All-cause medical costs of $7,708 (SE 81.9, p<0.001), $7,261 (SE 75.5, p<0.001) and $6,709 (SE 38.3) for MPR of 0–59%, 60–79% and 80% or more, respectively.
				All other prescription costs of $2,906 (SE 14.9, p<0.001), $2,684 (SE 13.7, not significant) and $2,651 (SE 7.0) for MPR of 0–59%, 60–79% and 80% or more, respectively.
				Statin prescription costs of $488 (SE 2.2, p<0.001), $664 (SE 2.0, p<0.001) and $838 (SE 1.0) for MPR of 0–59%, 60–79% and 80% or more, respectively.
Stuart et al. (2011) [63]	GLM with gamma distribution and log link	Adherence is measured using pill counts during entire follow-up period, and defined as a variant of MPR—the number of pills aggregated into 30-pill fills, divided by the number of months observed for each study subject (up to 36 months).	Median 3-year adherence was 77%.	$832 (SE 219, p<0.01) or 2.1% lower annual Medicare expenditure for 10% more adherent patients.
Wu et al. (2011) [58]	Logistic regression model and multiple-linear regression model with natural logarithm	Adherence is defined as 1-year MPR ≥ 80%.	37% were adherent.	(Reporting for adherent vs non-adherent patients)
				Lower ED visits (OR 0.71 [0.519, 0.812], p<0.01).
				Lower hospitalisations (OR 0.80 [0.636, 0.966], p<0.05).
				Lower all-cause medical cost (estimated coefficient -0.14 with SE 0.0638, p<0.05).
				Lower hyperlipidaemia-related cost (estimated coefficient -0.11 with SE 0.07, p<0.05).
Chen et al. (2012) [59]	Logistic regression model	Adherence is defined as supply for statins ≥ 90 days in the year prior to hospitalisation.	45% were adherent.	Lower risk of hospital readmission (OR 0.91 [0.85, 0.97], p<0.01) for adherent patients.
Roberts et al. (2014) [64]	Logistic regression model	6-month PDC ≥ 80%	61% were adherent.	(Reporting figures for adherent vs non-adherent patients)
				Less likelihood ED visit (OR of 0.86 [0.51, 1.43] at p>0.05) and
				less likelihood of hospitalisation (OR of 0.85 [0.48, 1.52] at p>0.05).
Zhao et al. (2014) [60]	GLM with gamma distribution and log link function and logistic regression model	Eight categories of 12-month MPR, <40%, 40%-59%, 60%-69%, 70%-79%, 80%-84%, 85%-89%, 90%-95%, and 96%-100%.	6%, 69%, 3%, 4%, 3%, 2%, 6% and 6% were in adherence categories of <40%, 40%-59%, 60%-69%, 70%-79%, 80%-84%, 85%-89%, 90%-95%, and 96%-100%, respectively.	(Compared to MPR<40%)
				Greater healthcare costs as much as CR 1.074 [1.011, 1.140] at p = 0.02, CR 1.140 [1.057, 1.229] at p = 0.001, CR 1.112 [1.031, 1.199] at p = 0.006, CR 1.186 [1.091, 1.288] at p = 0.001, CR 1.209 [1.136, 1.286] at p<0.001 and CR 1.188 [1.123, 1.256] at p<0.001) for the adherence categories of 40%-59%, 60%-69%, 80%-84%, 85%-89%, 90%-95%, and 96%-100%, respectively (No significant difference for adherence 70%-79% at p = 0.199).
				No significant difference in all-cause hospitalisations (p>0.05).
				Lower ED visits as much as ED visit ratio of 0.656 [0.524, 0.821] at p<0.001, 0.643 [0.512, 0.807] at p<0.001, 0.722 [0.569, 0.916] at p = 0.007, 0.651 [0.544, 0.779] at p<0.001 and 0.637 [0.544, 0.747] at p<0.001) for the adherence categories of 60%-69%, 80%-84%, 85%-89%, 90%-95%, and 96%-100%, respectively (No significant difference for adherence categories, 40%-59% and 70%-79% at p>0.2).
Li and Huang (2015) [62]	Logistic and linear regression models	Adherence is defined as MPR during entire follow-up period ≥ 80%.	59% were adherent.	(Reporting for adherent vs non-adherent patients)
				Lower all-cause hospitalisations (OR 0.32 [0.30, 0.35], p<0.001).
				No significant difference in coronary artery disease (CAD) hospitalisations and ED visits (p = 0.16 and 0.59, respectively.
				Lower total hospitalisation expenditure (-16,247.12 [-17,174.97, -15,319.26], p<0.001).
				Additional information ^a^
				Mean total hospitalisation expenditure is 130,905.90.
Mehta et al. (2019) [55]	Cox proportional hazards model	1. 12-month PDC.	Average PDC: 72–83%	Statin PDC was associated, not significantly, with lower risk of all-cause readmission (HR 0.832 [0.568, 1.219], p>0.1)).
		2. GlowCap adherence (GC), the number of days the pill bottle was opened divided by the total		
		number of days followed.	Average GC: 68–89%	Statin GC was associated with lower risk of all-cause readmission (HR 0.663 [0.467, 0.940], p<0.05).
Kirsch et al. (2020) [65]	Generalized additive mixed model	1-year PDC for each year in follow-up period.	(For the first, second and third year)	The impacts of PDC on several cost outcomes were graphically shown in the study, separately for male and female patients.
			Average PDC for male: 84%, 83% and 81%.	The Impacts on healthcare cost, ambulatory cost, hospitalisation cost, and remedy and aid cost for both male and female patients were found insignificant.
			Average PDC for female: 75%, 74% and 72%.	
				The impact on medication cost for male patients and impact on rehabilitation cost for female patients were found insignificant.

				Non-linear relationship between PDC and medication cost for female patients was found (p = 0.0001), showing peaks for the cost at very low, around 55% and very high PDCs.
				Positive relationship between PDC and rehabilitation cost for male patients was found (p = 0.0129).

a. Additional information is provided for comparison purpose when the reported figures are not in relative terms.

CHD = coronary heart disease; CR = cost ratio; ED = emergency department; GLM = generalized linear model; HR = hazard ratio; HRUHC = healthcare resource utilisations and healthcare costs; MAP = medication adherence or persistence; MPR = medication possession ratio; OR = odd ratio; PDC = proportion of days covered; RR = relative risk; SD = standard deviation; and SE = standard error

**Table 9:** Direction of impact of suboptimal adherence or persistence on healthcare resource utilisations and healthcare costs.

	Studies on antidepressants										Studies on bisphosphonates							Studies on statins												
	Eaddy et al. (2005) [46]	Katon et al. (2005) [49]	Cantrell et al. (2006) [40]	Robinson et al. (2006) [43]	Stein et al. (2006) [68]	Tournier et al. (2009) [48]	Ereshefsky et al. (2010) [66]	Albrecht et al. (2017) [45]	Vega et al. (2017) [44]	Aznar-Lou et al. (2018) [47]	Briesacher et al. (2007) [50]	Sunyecz et al. (2008) [41]	Eisenberg et al. (2015) [52]	LaFleur et al. (2015) [70]	Ferguson et al. (2016) [69]	Kjellberg et al. (2016) [53]	Sharman Moser et al. (2016) [51]	Cheng et al. (2006) [54]	Gibson et al. (2006) [56]	Stuart et al. (2009) [42]	Aubert et al. (2010) [57]	Pittman et al. (2011) [61]	Stuart et al. (2011) [63]	Wu et al. (2011) [58]	Chen et al. (2012) [59]	Roberts et al. (2014) [64]	Zhao et al. (2014) [60]	Li and Huang (2015) [62]	Mehta et al. (2019) [55]	Kirsch et al. (2020) [65]
Total healthcare cost	+-			-	X		+		+		+	+	X	+		+	+	X	X		+	+	+				-			X
DS healthcare cost				-									+	-		+								+						
Medical cost excluding pharmacy cost	+-	+	+		+-				+							+				+	+	+		+						
DS medical cost excluding pharmacy cost																+						+								
Hospitalisation cost	X	+									+																	+		X
DS hospitalisation cost						X																								
Non-DS hospitalisation cost						+																								
ED cost	X																													
Outpatient cost	X	+									+																			X
General practice service cost	X					-																								
Rehabilitation cost																														+-
Remedy and aid cost																														X
Pharmacy cost					-	-			-		-					-			+-											+-
DS Pharmacy cost	X													-		-						-								
Non-DS Pharmacy cost	X																					+								
Hospitalisations								X				+				+			+-	+	+			+	+	X	X	+	+-	
Hospital days																				+										
DS hospitalisations																+			+			+						X		
ED visits								X								+			+-					+		X	+	X		
DS ED visits																														
Outpatient visits												-				+														
DS Outpatient visits																+														
General practice visits										+									+-											
Specialty visits										X																				
DS specialty visit						+																								
Non-DS specialty visit						+																								
Combined HRUHC															+															

+: Greater for nonadherent or nonpersistent patients

-: Lower for nonadherent or nonpersistent patients

X: Not significant result (using measure of significance as defined in the paper)

+-: Mixed results from use of multiple measures of MAP

ED: Emergency departmentDS: Disease-specific. i.e., depression-related, osteoporosis-related and cardiovascular disease-related for antidepressants, bisphosphonates and statins, respectively

Medical cost: Healthcare cost not including pharmacy cost

### Quality assessment

We used the Quality Assessment Tool for Observational Cohort and Cross-Sectional Studies [34] to assign each study a grade of either good, fair or poor. Of the 30 studies, 27 achieved a good rating while three studies achieved a fair rating primarily due to not reporting detailed statistical results for the total healthcare cost including pharmacy costs [40], not reporting detailed statistical results for hospitalisations and outpatient visits [41], and not reporting clear definitions of MAP and HRUHC measures [42]. The assessments are summarised in Table 10.

**Table 10:** Quality assessment of reviewed studies using the Quality Assessment Tool for Observational Cohort and Cross-Sectional Studies.

	Studies on antidepressants										Studies on bisphosphonates							Studies on statins												
	Eaddy et al. (2005) [46]	Katon et al. (2005) [49]	Cantrell et al. (2006) [40]	Robinson et al. (2006) [43]	Stein et al. (2006) [68]	Tournier et al. (2009) [48]	Ereshefsky et al. (2010) [66]	Albrecht et al. (2017) [45]	Vega et al. (2017) [44]	Aznar-Lou et al. (2018) [47]	Briesacher et al. (2007) [50]	Sunyecz et al. (2008) [41]	Eisenberg et al. (2015) [52]	LaFleur et al. (2015) [70]	Ferguson et al. (2016) [69]	Kjellberg et al. (2016) [53]	Sharman Moser et al. (2016) [51]	Cheng et al. (2006) [54]	Gibson et al. (2006) [56]	Stuart et al. (2009) [42]	Aubert et al. (2010) [57]	Pittman et al. (2011) [61]	Stuart et al. (2011) [63]	Wu et al. (2011) [58]	Chen et al. (2012) [59]	Roberts et al. (2014) [64]	Zhao et al. (2014) [60]	Li and Huang (2015) [62]	Mehta et al. (2019) [55]	Kirsch et al. (2020) [65]
1. Was the research question or objective in this paper clearly stated?	Y	Y	Y	Y	Y	Y	Y	Y	Y	Y	Y	Y	Y	Y	Y	Y	Y	Y	Y	Y	Y	Y	Y	Y	Y	Y	Y	Y	Y	Y
2. Was the study population clearly specified and defined?	Y	Y	Y	Y	Y	Y	Y	Y	Y	Y	Y	Y	Y	Y	Y	Y	Y	Y	Y	Y	Y	Y	Y	Y	Y	Y	Y	Y	Y	Y
3. Was the participation rate of eligible persons at least 50%?	-	-	-	-	-	-	-	-	-	-	-	-	-	-	-	-	-	-	-	-	-	-	-	-	-	-	-	-	-	-
4. Were all the subjects selected or recruited from the same or similar populations (including the same time period)? Were inclusion and exclusion criteria for being in the study prespecified and applied uniformly to all participants?	Y	Y	Y	Y	Y	Y	Y	Y	Y	Y	Y	Y	Y	Y	Y	Y	Y	Y	Y	Y	Y	Y	Y	Y	Y	Y	Y	Y	Y	Y
5. Was a sample size justification, power description, or variance and effect estimates provided?	Y	Y	Y	Y	Y	Y	Y	Y	Y	Y	Y	Y	Y	Y	Y	Y	Y	Y	Y	Y	Y	Y	Y	Y	Y	Y	Y	Y	Y	Y
6. For the analyses in this paper, were the exposure(s) of interest measured prior to the outcome(s) being measured?	N	N	N	NR	N	N	N	NR	Y	NR	N	N	Y	N	N	Y	Y	N	Y	N	Y	N	N	N	Y	N	N	N	Y	N
7. Was the timeframe sufficient so that one could reasonably expect to see an association between exposure and outcome if it existed?	Y	Y	Y	Y	Y	Y	Y	Y	Y	Y	Y	Y	Y	Y	Y	Y	Y	Y	Y	Y	Y	Y	Y	Y	Y	Y	Y	Y	Y	Y
8. For exposures that can vary in amount or level, did the study examine different levels of the exposure as related to the outcome (e.g., categories of exposure, or exposure measured as continuous variable)?	Y	Y	Y	Y	Y	Y	Y	Y	Y	Y	Y	Y	Y	Y	Y	Y	Y	Y	Y	Y	Y	Y	Y	Y	Y	Y	Y	Y	Y	Y
9. Were the exposure measures (independent variables) clearly defined, valid, reliable, and implemented consistently across all study participants?	Y	Y	Y	Y	Y	Y	Y	Y	Y	Y	Y	Y	Y	Y	Y	Y	Y	Y	Y	N	Y	Y	Y	Y	Y	Y	Y	Y	Y	Y
10. Was the exposure(s) assessed more than once over time?	N	N	N	N	N	N	N	Y	N	N	N	N	N	N	N	N	N	N	N	N	N	N	N	N	N	N	N	N	N	Y
11. Were the outcome measures (dependent variables) clearly defined, valid, reliable, and implemented consistently across all study participants?	Y	Y	Y	Y	Y	Y	Y	Y	Y	Y	Y	Y	Y	Y	Y	Y	Y	Y	Y	N	Y	Y	Y	Y	Y	Y	Y	Y	Y	Y
12. Were the outcome assessors blinded to the exposure status of participants?	-	-	-	-	-	-	-	-	-	-	-	-	-	-	-	-	-	-	-	-	-	-	-	-	-	-	-	-	-	-
13. Was loss to follow-up after baseline 20% or less?	-	-	-	-	-	-	-	-	-	-	-	-	-	-	-	-	-	-	-	-	-	-	-	-	-	-	-	-	-	-
14. Were key potential confounding variables measured and adjusted statistically for their impact on the relationship between exposure(s) and outcome(s)?	Y	Y	N	Y	Y	Y	Y	Y	Y	Y	Y	N	Y	Y	Y	Y	Y	Y	Y	Y	Y	Y	Y	Y	Y	Y	Y	Y	Y	Y
**Number of N or NR**	**2**	**2**	**3**	**2**	**2**	**2**	**2**	**1**	**1**	**2**	**2**	**3**	**1**	**2**	**2**	**1**	**1**	**2**	**1**	**4**	**1**	**2**	**2**	**2**	**1**	**2**	**2**	**2**	**1**	**1**
**Quality rating** ^**a**^	**G**	**G**	**F**	**G**	**G**	**G**	**G**	**G**	**G**	**G**	**G**	**F**	**G**	**G**	**G**	**G**	**G**	**G**	**G**	**F**	**G**	**G**	**G**	**G**	**G**	**G**	**G**	**G**	**G**	**G**

Y: Yes; N: No; NR: Not reported; -: NA, G: Good; F: Fair; P: Poor

a. Rating is done by giving good for 0–2 N or NR, fair for 3–4 N or NR and poor for greater than 4 N or NR.

### Descriptive results

#### Study characteristics

*Antidepressants*. Ten studies reviewed considered the impact of MAP to antidepressants on HRUHC. These were all retrospective cohort studies using health research or claims database. Eight studies were conducted in the US. The studies were conducted with the data covering 3.6 years on average with standard deviation (SD) of 1.3 years, between 1999 and 2014. Three studies [43–45] were on all classes of antidepressants, two studies [46, 47] were on all selective serotonin reuptake inhibitors (SSRIs) medications, and other studies were on selected classes of antidepressants.

Seven studies broke down the examination period into three periods, commonly used in MAP research. The three periods are the baseline period in which patient characteristics are measured, the MAP period in which MAP is measured, and the follow-up period in which outcomes are measured. While lengths of each period are the same for each individual within a study, the date at which each period starts or ends depended on an individual date for the start of therapy, or index, which is screened in a pre-specified index period.

Cohort sizes ranged from 1,361 to 79,642 with an average of 31,659 (SD 25,382). All studies included both genders. Most studies targeted adult patients aged at least 18 years while one study was without age restriction [45], one study was for seniors aged 66 years or greater [48], and one study was for working-age patients aged between 16 and 65 [47]. Seven studies targeted patients with depression, and three other studies targeted patients with specified diseases other than depression but taking antidepressants: type-2 diabetes [44]; coronary artery disease, dyslipidaemia or diabetes [49]; and chronic obstructive pulmonary disease [45].

*Bisphosphonates*. Seven studies reviewed considered the impact of MAP to bisphosphonates on HRUHC: all were retrospective cohort studies using health research or claims database. Four studies were conducted in the US. The studies were conducted with the data covering 8.0 years on average (SD 1.8 years), between 1999 and 2013. Five studies broke down the examination period into baseline, MAP, and follow-up period. All studies considered alendronate and risedronate either exclusively [41, 50, 51] or as part of a wider medication range. Cohort sizes ranged between 17,770 and 38,234 with an average of 29,544 (SD 7,974). Most studies targeted female patients except two studies that considered both male and female [50, 52]. All studies set a minimum age to be included in the study cohort, the most common being 55 years [51–53]. No studies limited the cohort to those with specific underlying diseases other than osteoporosis.

*Statins*. There were 13 studies reviewed that considered the impact of MAP to statins on HRUHC. These studies were retrospective cohort studies using health research or claims data except for a prospective observational study [54], a longitudinal study using a survey [63], and a secondary analysis using a randomised clinical trial [55]. Ten studies were conducted in the US. The studies were conducted with data covering 4.3 years on average (SD 2.6 years) between 1997 and 2015. Four studies divided the examination period into baseline, MAP and follow-up period.

Five studies were on all classes of statins [56–60], three studies were on selected classes of statins [54, 61, 62], and five studies were on several medications including statins [42, 55, 63–65]. All studies were conducted with relatively large cohort sizes of over 500 individuals (between 682 and 381,422), except Cheng, Chan [54], which included 83 individuals. The average sample size was 44,744 (SD 101,804). Five studies [54, 56–59] targeted all adult patients and four did not specify an age range [42, 63–65], while other studies were on different age ranges, as seen in Table 5. Six studies were for patients with baseline diseases including diabetes [42, 58, 63], coronary heart disease [54], and acute myocardial infarction [55, 65]; the remaining studies were for any patients newly prescribed statins.

#### Medication adherence or persistence

*Antidepressants*. While various methods were used to measure MAP to antidepressants, the most common measure was persistence, defined as the duration of time from initiation to discontinuation of therapy. Five studies used it as a single method [48, 66], as one of several methods [40, 44], or in combination with another method (for an overall MAP measure) [49]. Discontinuation was defined by having a treatment gap greater than 15 days [40, 44, 49], or greater than 30 days [48, 66].

Medication possession ratio (MPR), calculated by adding the days’ supply for all medications and then dividing over a set period [67], was used by four studies, either as a single measure [68], as one of several measures [40, 44] or in combination with a persistence measure [49]. Proportion of days covered (PDC), the proportion of days a patient has a drug administered in a study interval [67], was used by one study [45]. For MPR and PDC measures, the measured adherence was categorised to form an independent variable in the studies, the most common method being categorising MPR or PDC of at least 80% as adherent [40, 44, 49, 68]. Other methods used include nonadherence for the first prescription [47] and customised rules [43, 46].

Nine studies measured MAP for a fixed duration of six months or 180 days [40, 44, 48, 49, 66], of one year [46, 68], of one month [47] and of 214 days [43]. One study measured MAP during the available follow-up period for each patient [45].

Reported average values of MAP ranged between 19% and 85%. However, heterogeneity in the length of MAP period and type of MAP measure used did not allow an estimation of an aggregate average.

*Bisphosphonates*. For bisphosphonate studies, MPR was used to measure MAP by six studies, as a single measure [50, 52, 53], in combination with a persistence measure [51, 69], or in addition to a persistence measure [41].

Of those, four studies defined adherence as MPR at least either 70% [51–53] or 80% [41]. One study categorised MPR into several groups by threshold [50] and one study did not form categories but reported a proportion of patients who achieved at least 80% of MPR [69]. The studies using persistence measures defined discontinuation as a treatment gap greater than 30 days [41], 60 days [51], or three months [69]. One study that did not use MPR categorised longitudinal quarterly MAP into four categories: non-switching, switching, discontinuing, and reinitiating [70].

Three studies measured MAP for a one-year period [51–53]. Other studies measured MAP during the available period for each patient. The wide range of reported average values of MAP between 20% and 85% could not be summarised further because of the heterogeneity in the length of MAP period and type of MAP measure used.

*Statins*. Seven statin studies used MPR as a single measure of MAP by defining MAP as an MPR of at least 80% [56–58, 62], by having multiple categories of MAP using the MPR [60, 61], or by using the MPR as a numerical variable [63]. Three studies used PDC by using the threshold at 80% to define MAP for multiple medications [64], as a numerical variable [65], and as a numerical variable along with GlowCap adherence measure, the number of days the electronic pill bottle was opened divided by the total number of days followed [55]. Other methods include assessment based on percentage of doses taken within the suggested time interval and without time constraint [54], statin supply for at least 90 days in the year prior to hospitalisation [59], and annual number of prescription fills [42].

Ten studies measured MAP for fixed duration of one year [55, 58, 60, 61, 65], of six months [54, 59, 64], and of 18 months to two years [56, 57]. Other studies measured MAP for the available period for each patient. The wide range of reported average values of MAP between 17% and 96% could not be summarised further because of the heterogeneity in the length of MAP period and type of MAP measure used.

#### Impact of MAP on HRUHC

*Antidepressants*. Among the ten studies on antidepressants, six reported significantly increased HRUHC by non-MAP, including total healthcare cost [44, 66], medical cost excluding pharmacy cost [40, 44, 49], hospitalisation cost [49], outpatient cost [49], cost of non-psychiatric hospitalisation [48], psychiatric and other specialty visits [48] and general practice (GP) visits [47].

Three studies reported reduced pharmacy costs [44, 48, 68]. Some studies also found reduced total and mental health specific healthcare costs [43], reduced cost of GP services [48], mixed results for total healthcare cost and medical cost excluding pharmacy cost [46], and mixed results for medical cost excluding pharmacy cost [68] over several categories of MAP. Insignificant impacts were reported on total healthcare cost [68], mental health specific hospitalisation cost [48], hospitalisations [45], ED visits [45] and specialty visits [47]. In addition, insignificant impacts of MAP on hospitalisation cost, ED cost, outpatient cost, GP service cost, and antidepressant and other pharmacy costs were reported [46].

*Bisphosphonates*. All seven studies on bisphosphonates reported significantly increased utilisation or cost of at least one type of health resource following non-MAP, including total healthcare cost [41, 50, 51, 53, 70], osteoporosis-related healthcare cost [52, 53], all-cause combined and osteoporosis-related medical cost excluding pharmacy cost [53], hospitalisation cost [50], outpatient cost [50], outpatient visits and use of ED services [53], hospitalisations [41, 53], osteoporosis-related hospitalisations and outpatient services [53], and combined HRUHC [69].

Four studies reported reduced utilisation or cost of several types of resource, including pharmacy cost [50, 53], cost of bisphosphonates [53, 70], osteoporosis-related healthcare cost [70], and outpatient visits [41].

*Statins*. Nine of 13 statin studies reported that at least one type of HRUHC significantly increased following non-MAP. The increased HRUHCs include total healthcare cost [57, 61, 63], CVD-related healthcare cost [58], medical cost excluding pharmacy cost [42, 57, 58, 61], CVD-related medical cost excluding pharmacy cost [61], pharmacy cost other than statins [61], hospitalisation cost [62], ED visits [58, 60], hospitalisations [42, 57–59, 62], hospital days [42], and CVD hospitalisations [56, 61]. In contrast, Zhao, Zabriski [60] found decreased total healthcare costs for nonadherent patients. Pittman, Chen [61] found lower statin prescription cost for nonadherent patients.

Several studies found insignificant impacts of MAP to statins on total healthcare cost [54, 56, 65], hospitalisation cost [65], outpatient cost [65], remedy and aid cost [65], hospitalisations [60, 64], CVD hospitalisations [62], and ED visits [62, 64]. Gibson, Mark [56] divided patients into two groups–new users and continuing users–and found mixed results for pharmacy cost, GP visits, ED visits, and hospitalisations. Kirsch, Becker [65] found non-linear impact on pharmacy cost only for female patients and positive impact on rehabilitation cost only for male patients. Mehta, Asch [55] used multiple measures of MAP and found mixed results on hospitalisation.

#### Aggregate data meta-analysis

We conducted a meta-analysis to estimate the impact of MAP on HRUHC when reported for at least two different population cohorts using comparable measures of MAP to the same medication class and the same type of HRUHC. Of 30 studies, only eight were used in the meta-analysis to obtain five synthesised results. Further findings were not possible due to the heterogenous types of MAP and HRUHC examined by the reviewed studies.

Table 11 summarises the averaged impacts of MAP on HRUHC from the meta-analysis. Forest plots and more detailed figures are found in S2 File. For antidepressants, having greater than 80% adherence by either medication possession ratio (MPR) or proportion of days covered (PDC) during a six-month or 180-day period was found to reduce the total medical cost not including pharmacy cost by 12% [-16%, -8%], the hospitalisation cost by 26% [-48%, 4%] and outpatient cost by 10% [-17%, -2%]. The impact of persistence in using antidepressants for a 180-day period without 15-day gap on total healthcare cost was found insignificant. For bisphosphonates, having greater than 70% or 80% adherence by one-year MPR was found to reduce the total healthcare cost by 3% [-6%, 0%]. For statins, no meta-analysis could be performed mainly due to inconsistency in the measures of MAP within studies.

**Table 11:** Result of meta-analysis.

Type of HRUHC	Measure of MAP	Number of cohorts^a^	N	Difference in cost	p-value
				% [95% CI]	
***Antidepressants***					
**Total medical cost excluding pharmacy cost**	MPR or PDC ≥ 80% during 6-month or 180-day period	5	34,074	-12% [-16%, -8%]	< 0.00001
**Hospitalisation cost**	6-month MPR ≥ 80%	3	9,766	-26% [-48%, -4%]	0.02
**Outpatient cost**	6-month MPR ≥ 80%	3	9,766	-10% [-17%, -2%]	0.01
**Total healthcare cost**	Absence of 15-day gap during 180-day period	2	46,842	-1% [-8%, 6%]	0.80
***Bisphosphonates***					
**Total healthcare cost**	MPR ≥ 70% during 1-year period	3	83,909	-3% [-6%, 0%]	0.07

a. Note three cohorts from a single study (Katon et al., [49]) were used for the calculation of impact of MAP to antidepressants on total medical cost excluding pharmacy cost, hospitalisation cost and outpatient cost.

## Discussion

This review provides a comprehensive view on the impact of MAP to antidepressants, bisphosphonates and statins on HRUHC. It is the first to provide an integrated understanding of the impact of MAP to three medication classes on HRUHC using a broad array of HRUHC measures including total healthcare cost, disease-specific healthcare cost, medical cost excluding pharmacy cost, pharmacy cost, cost of medication, hospitalisation, outpatient service use, ED visits, GP visits and specialty visits. Previous reviews on the impact of MAP to these three medication classes on HRUHC did not address diverse measures of MAP or HRUHC, or did not make comparisons with other medication classes used for prevalent conditions [e.g., 6, 71, 72].

MAP varies by medication class, MAP measure, length of follow-up period and study design, limiting scope for comparison. Nevertheless, the review found broad consistency in reported MAP to antidepressants, with the percentage of patients with MPR or PDC at least 80% during a 180 day or six month period ranging between 36% and 43% [40, 44, 49, 68] and percentages of persistent patients as measured by continued use in 180 days without a 30-day gap of 19% [66] and 35% to 47% [48]. These findings are comparable with a previous review (on studies not limited to those on the impact of MAP on HRUHC) indicating that 35% to 55% of patients remain adherent or persistent to antidepressant therapy at six months [73].

The proportion of patients adherent to bisphosphonates were between 20% and 70% in the studies that measured MAP as a MPR or PDC of at least 70% or 80% during a one year period [50–53]. The mean persistence of oral bisphosphonates for one year similarly ranged between 18% and 75% in a systematic review by Fatoye, Smith [74]. The wide range of MAP could be partly due to variance between studies in factors related to non-MAP including younger age [74] and more frequent dosing [74, 75]. Of the four studies compared above [50–53], the lower bound of the range (i.e., 20%) was found from Briesacher, Andrade [50] on patients aged at least 40, compared with the other three studies which were all on patients aged at least 55.

Among statin users, 17% to 68% were found to have a MPR at least 80% during a one year period [58, 60, 61]. These findings are comparable with a previous review showing that patients with a MPR for statins at least 80% ranged between 18% and 92% for different lengths of MAP period [76]. Consistent with previous studies that found lower MAP for new statin users compared to continuous users [56, 76], Wu, Seiber [58] and Zhao, Zabriski [60] reported a lower percentage of adherence for new statin users than Pittman, Chen [61] for continuous users.

The MPR and PDC were the most frequently used methods to summarise MAP. While they both measure the percentage of the time that a patient has medication available, the PDC was introduced to mitigate the overestimation problem of the MPR in which early refill (i.e. refill when the medication is still available) is included in the amount of medication available in the measuring period [77]. We found the tendency that the PDC measure was used for studies published later.

Several studies [40, 41, 44, 45, 54, 55] reported results that allow comparison among multiple methods of measuring MAP. For example, Mehta, Asch [55] compared MAP as measured by PDC based on pharmacy claims as well as GlowCap adherence using electronic pill bottles. They found that the significantly lower risk of all-cause readmission of patients previously discharged with a diagnosis of acute myocardial infarction was found only when the GlowCap adherence was used, highlighting the importance of measurement method for MAP.

In general, non-MAP was found to be associated with increased HRUHC. Of the 30 papers included, 25 found a significant positive association between non-MAP and one or more measures of HRUHC, although in some cases negative or mixed associations were found for other HRUHC measures. This generally positive association between non-MAP and HRUHC has also been found in previous reviews [e.g. 6, 78].

This review revealed that the association between non-MAP and increased healthcare costs is most definitive for medical costs excluding pharmacy costs. Of 10 papers that assessed total healthcare costs net of pharmacy costs, eight reported a positive association with non-MAP, the remaining two reporting mixed results. Non-MAP was found to be negatively associated with total pharmacy costs for five of the seven papers that assessed this, reflecting higher pharmacy costs for patients that are adhering to, and therefore consuming more of, their medication. Of the 18 papers that assessed total healthcare costs, 10 reported a positive association between total healthcare costs and non-MAP, and a further six found no significant association or mixed results across multiple MAP categories. This result is a combination of generally lower pharmacy costs and generally higher medical costs excluding pharmacy costs for non-MAP patients. These findings related to healthcare and pharmacy costs apply across all three medications considered.

Increase in hospitalisations and ED visits associated with non-MAP was frequently reported. Of 12 studies that measured the impact of non-MAP on hospitalisation, seven found a positive impact while the rest found either mixed or insignificant results. Of seven studies that assessed the impact of non-MAP on ED visits, three found a positive impact while four found insignificant results or mixed results across multiple patient categories. Several HRUHC measures were reported by too few papers to enable comparison; for example, the impact on specialty visits was reported by only one study [47].

While general patterns of resource use were similar across the three medication classes, some differences exist. First, the pattern of increased healthcare cost following non-MAP was the most apparent in bisphosphonate studies. All seven studies on bisphosphonates found an increase in at least one type of HRUHC. Of six studies that assessed the total healthcare cost, five found a positive association with non-MAP, while one found no significant association. The clearer pattern found could be related to the tendency that studies on bisphosphonates used longer data periods (average 8.0 years compared to average 3.6 and 4.3 years for the studies on antidepressants and statins, respectively) and less heterogenous cohorts (typically females aged at least 55 compared to all adult patients in the studies on antidepressants and statins).

Second, the impacts of non-MAP on hospitalisation and ED visits were more frequently studied on statins and were found to be generally positively associated. Third, the associations between non-MAP and total pharmacy costs were negative for all studies on antidepressants and bisphosphonates that assessed this, but were heterogenous for the studies on statins; negative for continuing users [56], non-linear for female patients [65] and insignificant for others. This shows that non-MAP to statins does not necessarily imply non-MAP to other medications. Future studies to examine such selective non-MAPs will be useful to further understand the reasons for non-MAP to statins.

Last, in the studies on antidepressants and statins compared to bisphosphonates, notwithstanding the general finding of positive associations, there were greater numbers of reported insignificant impacts of non-MAP on HRUHC. The impacts of non-MAP may not be sufficiently captured with a short follow-up period given that nine [45–48, 54, 60, 64, 65, 68] of 11 studies that reported insignificant associations measured MAP and HRUHC for the same (i.e., overlapping) six-month to one-year period. There could also be idiosyncratic healthcare system factors. Li and Huang [62] found an insignificant impact on ED visits and discussed that the finding may not accurately show the impact on the occurrence of emergency situations that are potentially costly due to frequent non-emergency use of emergency care services in Taiwan.

Comparing the directions of impact on different types of HRUHC within an individual study can suggest underlying mechanisms linking MAP and HRUHC. For example, Tournier, Moride [48] found that persistence to antidepressants increases GP service costs and pharmacy costs but decreases specialty visits and non-psychiatric hospitalisation costs. This shows that spending on primary care to maintain mental health can reduce the costs associated with adverse health outcomes. Another similar pattern was found by Sunyecz, Mucha [41] reporting that persistence to bisphosphonates reduces total healthcare cost and hospitalisations but increases outpatient visits.

The meta-analysis found positive associations between non-MAP and HRUHC for total medical cost excluding pharmacy cost, hospital cost and outpatient cost for antidepressants all at 5% significance level, and total healthcare cost for bisphosphonates at 10% significance. The average magnitude of the impact of non-MAP to antidepressants on hospitalisation cost was estimated at 26%.

Heterogeneities in study location, data type, cost calculation method, HRUHC measure, analysis method and other characteristics limited the extent to which further meta-analysis could be conducted and overall conclusions drawn. This meant we could not conduct a meta-analysis on statins and a meta-analysis on other HRUHC components, and it limited the number of studies included in the meta-analyses that were conducted. Study characteristics will also influence the meta-analysis results. For example, more costly healthcare in the US than the UK [79] along with the preponderance of US studies included in the meta-analysis is expected to result in higher estimates of impact of non-MAP on HRUHC which may not be generalisable to non-US locations.

Comparisons across studies having different characteristics were not possible primarily due a limited number of studies having certain characteristics different to the majority. For example, a comparison by location was limited because the review included at most one study conducted in a non-US country for each medication class; a comparison by analysis method was limited because most studies used generalized linear models; and a comparison by data type was limited because most studies used an administrative dataset with limited use of other (e.g., survey data) types.

The quality assessments in Table 10 show that all included studies meet the majority of assessment criteria related to study objectives, selection criteria for study populations, justification of sample selection and size, measurement of MAP, study timeframes and quality of analysis. While 28 of 30 studies reviewed did not assess MAP more than once, a single MAP figure comprises multiple observations on medication use over time and hence any inaccuracy that may arise from assessing an exposure once only would be small. There was no evident difference in quality across the studies on the three medication classes.

Of the 30 reviewed studies, 27 studies used a large administrative dataset allowing for a sample size greater than 1,000 and measured MAP using administrative records of filling prescriptions (e.g., pharmacy claims). This approach to measuring MAP is standard and has the advantages that MAP is passively measured and easy to track for large populations [80] and does not influence participant behaviour. However, limitations include that filled medications are not necessarily taken; a diagnosis for which medications are prescribed is mostly unavailable; the reason for non-MAP is not known; a case of discontinuation recommended by health service provider is not identified (e.g., side effects); and data does not capture all medications used by a patient (e.g., data extracted from an insurance plan does not capture medications funded from other means). In addition, the measure of MAP can be sensitive to modelling decisions in preparing pharmacy data sets [81], and most reviewed studies did not provide details of these decisions.

Not measuring MAP prior to HRUHC was a common problem, most prominent in the studies on antidepressants. Although 22 studies set separate measurement periods for MAP and HRUHC, only eight clearly showed that MAP was measured strictly prior to HRUHC. As MAP could be affected directly by HRUHC (e.g., GP visits to get prescriptions) or indirectly by health conditions suggested by HRUHC (e.g., reassuring the need for medication at ED visit), measuring MAP prior to HRUHC would avoid potential reverse causality problems. The findings within these eight studies were generally consistent with the overall findings of this review.

Limited generalisability was found for several studies. For example, results within Gibson, Mark [56] may be only applicable for an insured population as it was based on patients covered by employer-sponsored health insurance. The patient population of Ferguson, Feudjo Tepie [69] is atypical in that a significant proportion had a high level of glucocorticoid use.

Many studies did not clearly specify the payer and recipient for the measured healthcare costs although such specification will be useful when study findings are used for developing health policy. Only a few studies specifically stated from whose perspective the costs are measured. For example, Cheng, Chan [54] reported that the costs were calculated for each patient from the perspective of a public health provider. Considering the type of data used by the majority (i.e., administrative claim data), most studies that assessed healthcare costs are likely to have measured the cost paid by an insurance company or the public healthcare system to healthcare service providers.

There are several limitations of this systematic review. First, only a limited meta-analysis could be undertaken due to heterogeneous types of MAP and HRUHC within the studies. There is a need for agreement on consistent methods to be applied to measure MAP and more studies on each type of HRUHC to improve comparability. Second, the review does not attempt to directly evaluate individual patient factors that may influence the impact of MAP on HRUHC (e.g., age, sex, comorbidity, severity of disease): such factors were highly heterogenous across different studies and could not be meaningfully incorporated. However, 28 of 30 studies did measure these and other potentially confounding variables and made statistical adjustments accordingly in measuring the impact of MAP on HRUHC, enabling valid comparison without a need for direct evaluation of these factors in this review.

Third, 22 of the studies were conducted in the US and therefore results may fail to reflect experience in other countries; this is due partly to the exclusion of non-English articles as well as to the preponderance of US studies. Several studies have found that exclusion of non-English articles is unlikely to result in bias [82, 83]. Among English articles, the strict search protocol limits bias in study selection and ensures that the US dominance is due to dominance of research. Additional research conducted outside the US will permit greater understanding of the impact of MAP on HRUHC dependent on healthcare systems.

Last, the review focuses only on HRUHC from a healthcare system perspective and does not address other non-healthcare burdens such as loss of productivity, absence from work, loss of quality of life and costs of home care or informal care. Several previous studies examined such burdens following non-MAP [84–87]. These aspects are outside the scope of our review however it should be acknowledged that total economic impact of non-MAP will be greater than that indicated by HRUHC within this review.

## Conclusions

This systematic literature review is the first to compare the impact of non-MAP to medications for three prevalent conditions—depression, osteoporosis and cardiovascular disease—on healthcare resource utilisation and cost. While previous reviews generally focused on finding the impact of MAP on particular healthcare costs or clinical outcomes, this review considered a wide range of measures and three different medication classes. From 30 included studies assessed to be of good or fair quality, we found generally positive associations between non-MAP and healthcare resource utilisation and cost for all three medication classes but most prominently for bisphosphonates. Notwithstanding this general finding, the significance and direction of associations was heterogenous across alternative HRUHC measures and medication classes. In some cases, non-MAP reduced healthcare resource utilisation or cost, particularly for pharmacy. The ability to quantitatively summarise the impact of non-MAP on healthcare resource utilisation and cost was challenged by a small number of studies reporting comparable results; the development of more consistent measures would enable more meaningful analysis. The study highlights the need to understand how and to what extent poor MAP exhausts healthcare resources to inform clinical practice, health policy and research.

## Supporting information

S1 Checklist PRISMA checklist. (DOCX)

S1 Fig Forest plots for aggregate meta-analysis. (DOCX)
