# Supplementary material for: A systematic review and meta-analysis on impact of suboptimal use of antidepressants, bisphosphonates, and statins on healthcare resource utilisation and healthcare cost
Source: PLoS One. 2022 Jun 29;17(6):e0269836. doi: 10.1371/journal.pone.0269836 (PMC9242484; doi:10.1371/journal.pone.0269836)
Supplement: S1 Fig — (DOCX) [file pone.0269836.s002.docx]

# Forest plots and detailed figures for meta-analysis

| ***Antidepressants*** |
| --- |
| **Total medical cost excluding pharmacy cost**  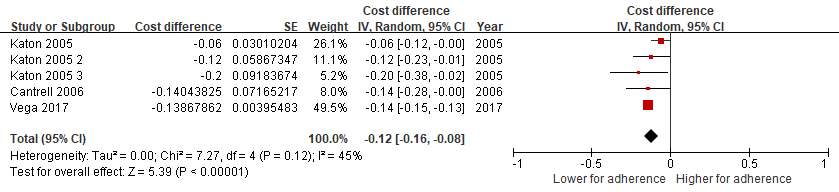  **Hospitalisation cost**  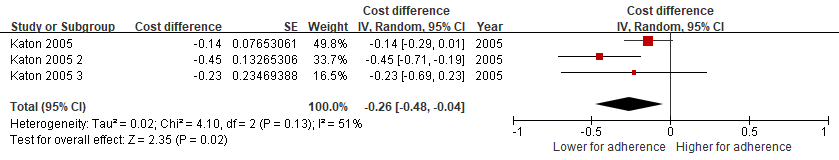  **Outpatient cost**  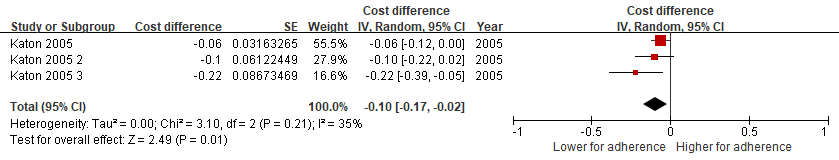  **Total healthcare cost**  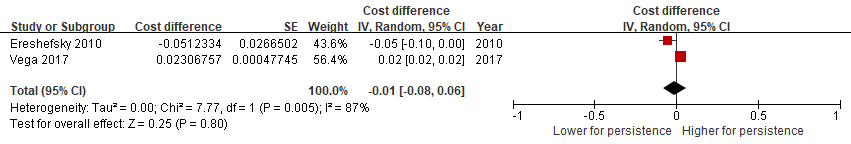 |
| ***Bisphosphonates*** |
| **Total healthcare cost**  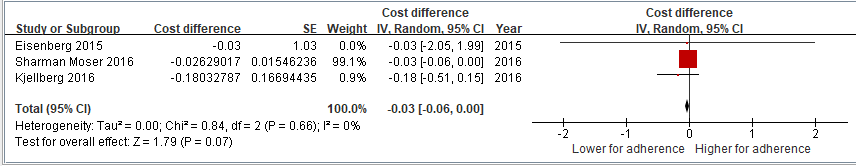 |
